# Dendritic Polyglycerol Sulfate for Therapy and Diagnostics

**DOI:** 10.3390/polym10060595

**Published:** 2018-05-29

**Authors:** Nadine Rades, Kai Licha, Rainer Haag

**Affiliations:** Institute for Chemistry and Biochemistry, Freie Universität Berlin, Takustraße 3, 14195 Berlin, Germany; nadine.rades@fu-berlin.de (N.R.); kai.licha@fu-berlin.de (K.L.)

**Keywords:** dendritic polyglycerol, heparin, inflammation, targeting

## Abstract

Dendritic polyglycerol sulfate (dPGS) has originally been investigated as an anticoagulant to potentially substitute for the natural glycosaminoglycan heparin. Compared to unfractionated heparin, dPGS possesses lower anticoagulant activity but a much higher anticomplementary effect. Since coagulation, complement activation, and inflammation are often present in the pathophysiology of numerous diseases, dPGS polymers with both anticoagulant and anticomplementary activities represent promising candidates for the development of polymeric drugs of nanosized architecture. In this review, we describe the nanomedical applications of dPGS based on its anti-inflammatory activity. Furthermore, the application of dPGS as a carrier molecule for diagnostic molecules and therapeutic drugs is reviewed, based on the ability to target tumors and localize in tumor cells. Finally, the application of dPGS for inhibition of virus infections is described.

## 1. Introduction

Within the last few decades, the development of polymeric therapeutics and diagnostics has attracted increasing interest within the research community. Many reviews have summarized these developments using synthetic polymers and biopolymers [1,2,3,4,5,6]. A polymer that plays a vital role in research on biocompatible polymers for nanomedical applications is dendritic polyglycerol (dPG). It is characterized by the combination of a stable, biocompatible polyether scaffold, high-end group functionality and a compact, well-defined dendrimer-like architecture [7]. Its applications range from protein resistant coatings to drug delivery systems, DNA-transfection agents, anticoagulating and anti-inflammatory drugs [8]. Sulfated dPG was investigated around 15 years ago to potentially substitute for heparin, but is also suitable for other applications due to its intrinsic properties.

**Figure 1:**
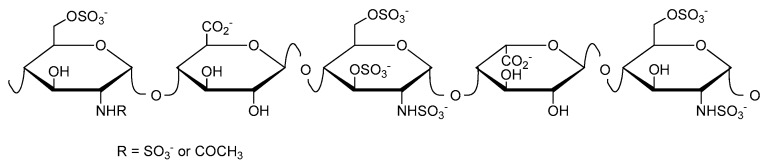
Schematic structure of heparin showing here the antithrombin-binding pentasaccharide sequence.

Heparin (Figure 1) was discovered in 1916, and has been used clinically as an anticoagulant since 1935 [9]. Further biological activities include the inhibition of complement activation [10,11], the inhibition of angiogenesis and tumor growth [12,13,14], and antiviral activity [15,16,17]. Heparin has been administered for the prevention and treatment of thromboembolic disorders for more than 80 years. However, it still has to be isolated from mammalian organs, implying the risk of disease transmission.

Other natural sulfated polysaccharides have the same biological activities as heparin, but also the same inherent risk of contamination with pathogens such as viruses and prions. Therefore, promising partially synthetic, sulfated linear polysaccharides as well as fully synthetic, sulfated linear polymers have been prepared [18,19]. Additionally, a polysulfated heparin mimetic based on branched polysaccharides, with much higher anticoagulation activity compared to the linear counterparts, has been described [20]. Avoiding the dependency on limited natural branched polysaccharides, Türk et al. published a synthetic approach to highly branched polysulfated heparin analogs based on dendritic polyglycerol [21]. Originally investigated as an agent with anticoagulant activity, dendritic polyglycerol sulfate (dPGS) showed high anti-inflammatory activity. Therefore, dPGS has additionally been examined for the diagnosis and therapy of inflammatory and related diseases. We have summarized these applications within this review.

## 2. Synthesis of dPGS

dPGS is synthesized by sulfation of dendritic polyglycerol. The first synthetic route of dPG was published by Sandler and Berg in 1966 using a variety of catalysts at room temperature [22]. Some years later, Dworak et al. demonstrated the first cationic ring opening polymerization of glycidol initiated by Lewis or Brønsted acids following two possible mechanisms: active chain end and activated monomer [23,24]. Since cationic polymerization processes are difficult to control due to multiple side reactions that hinder propagation including chain transfer and chain termination, only lower molecular weight dendritic polyglycerols (less than 10 kDa) with high polydispersity were obtained. The limitations of cationic polymerization of glycidol were overcome by employing an anionic ring-opening multi-branching polymerization (ROMBP) in combination with slow monomer addition described by Sunder et al. [25]. In this strategy, glycidol was added dropwise to a partially deprotonated initiator, namely, 1,1,1-tris(hydroxymethyl)propane (TMP), at elevated temperatures (90–100 °C) yielding low molecular weight dPG (*M*_n_ = 1.25–6.5 kDa) with narrow polydispersities (PDI < 1.5). The mechanistic details of ROMBP and improvements in dPG polymerization conditions were reviewed by Wilms et al. [26]. TMP is usually partially deprotonated (10%) with potassium methylate, and the resulting methanol is distilled off from the melt under reduced pressure to shift the reaction equilibrium to the side of the desired initiator which is subsequently dissolved in diglyme. The monomer glycidol is added slowly at elevated temperatures to minimize polymerization without initiator as well as cyclization. Due to fast proton exchange during the polymerization the different chain ends (secondary and primary alcohols) can grow simultaneously, resulting in a branched structure (DB = 0.5–0.7) [25,26,27]. Potential biomedical applications of dPG were reviewed by Calderon et al. in 2010 [8].

**Figure 2:**
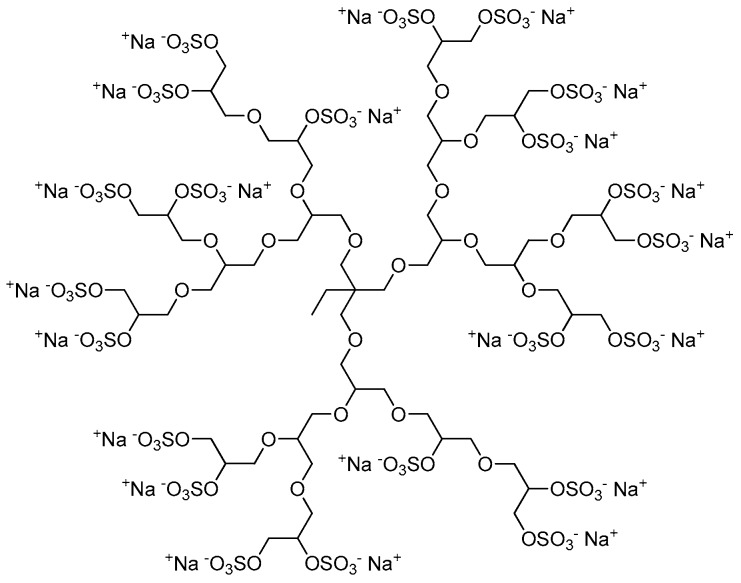
Representative and idealized structure of dendritic polyglycerol sulfate (dPGS).

The sulfation of dPG yielding polyanionic dPGS was first described by Türk et al. in 2004 [21]. Dendritic polyglycerols with three different molecular weights (*M*_n_ = 2.5, 5, and 10 kDa) were sulfated with sulfur trioxide-pyridine complex (SO_3_·py) according to a method described by Alban et al. [28]. A solution of SO_3_·py in *N*,*N*-dimethylformamide (DMF) was added dropwise to a solution of dPG in DMF over 4 h at 60 °C. The reaction mixture was stirred for additional 2 h at 60 °C and finally for 18 h at room temperature yielding negatively-charged dPGS with pyridinium as counter ions. By quenching the reaction with an aqueous sodium hydroxide solution to deprotonate the pyridinium ions, they were exchanged by sodium ions giving the desired product (Figure 2). To obtain dPGS carriers suitable for further chemical conjugation, azide or amine groups are usually introduced by mesylation and subsequent azidation of some hydroxy groups of the dPG, sulfation of the residual hydroxy groups, and finally reduction of the azide groups if necessary [29,30,31,32]. In most of the studies mentioned below, dPGS with a dPG core size of 6 kDa and a final molecular weight of around 12 kDa, respectively, was applied.

## 3. dPGS as an Anti-Inflammatory Agent

In 2004, Türk et al. published the synthesis of dPGS, which should serve as a highly branched polysulfated heparin analog based on dendritic polyglycerol [21]. The authors evaluated the anticoagulant and anticomplementary activities of dPGS in comparison to unfractionated heparin (UFH) using non-sulfated dPG as a control. dPG was inactive in all assays, showing that it had no influence on the coagulation and on the complement system. On the contrary, dPGS showed both anticoagulant as well as anticomplementary activity. Compared to UFH, dPGS possessed a significantly lower anticoagulant activity but much higher anticomplementary effect. The ratio of anticomplementary to anticoagulant activity was around two orders of magnitude higher for dPGS compared to UFH. Therefore, Türk et al. suggested that efficient anticomplementary doses could be combined with moderate anticoagulant activities to prevent bleeding, as could occur with UFH.

**Figure 3:**
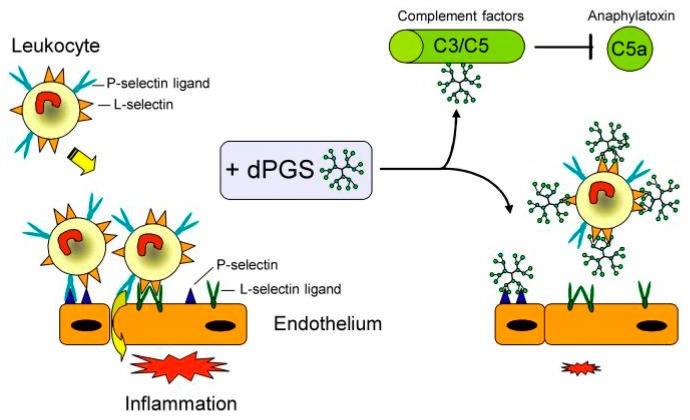
Pleiotropic anti-inflammatory effect of dPGS diminishes the inflammatory response and reduces leukocyte extravasation. Molecular targets of dPGS are the adhesion molecules L- and P-selectin. dPGS prevents leukocyte extravasation by shielding the selectins. Binding to complement factors C3 and C5 inhibits the formation of the proinflammatory anaphylatoxins. Reduction of the C5a level decreases vascular permeability and further leukocyte extravasation. By addressing these inflammatory targets simultaneously, dPGS promote the resolution of inflammation. Reprinted from [33].

In 2010, Dernedde et al. presented the multivalent selectin binding and the inhibition of complement activation by dPGS in vitro as well as the high anti-inflammatory effect of dPGS in vivo [33]. The authors assumed that polyanionic dPGS would strongly bind to the positively charged ligand binding pockets of endothelial P- and leukocytic L-selectin where usually the anionic sulfotyrosine residues of the physiological P-selectin glycoprotein ligand 1 (PSGL-1) bound. Besides P-selectin binding, PSGL-1 is also the most important L-selectin ligand in inflammatory settings [34]. Dernedde et al. prepared different dendritic polyglycerol sulfates with varying core size (2, 4, and 6 kDa) and degree of sulfation to obtain a detailed structure-activity relationship. They evaluated the selectin binding potential by a competitive surface plasmon resonance (SPR)-based binding assay. While E-selectin was not inhibited by dPGS, P- and L- selectin showed stronger binding of dPGS (in the nanomolar range) than UFH (in the micromolar range). A clear dependency on the size and degree of sulfation was observed; the larger the dPGS and the higher the degree of sulfation, the stronger was the binding inhibition. Moreover, their studies of selectin deficient mice indicated that the interaction of heparin with L- and P-selectin was necessary for its anti-inflammatory effect in vivo. Since dPGS had a higher binding affinity than heparin, Dernedde et al. speculated that dPGS was the more potent anti-inflammatory compound [33]. Furthermore, dPGS interacted with the complement factors C3 and C5 in vitro and reduced C5a levels in vivo in a mouse model of complement activation. Finally, the authors explained the decreased inflammatory response with a combination of different interactions of dPGS, namely, the binding and thus inhibition of L- and P-selectin causing leukocyte extravasation and of C5a generation causing leukocyte chemotaxis inhibition (Figure 3).

Further structure-activity studies were conducted by Haag and colleagues. By screening different dPG-based anions (dPG core size of 3 and 6 kDa), Weinhart et al. showed that these anions inhibited L-selectin with increasing efficiency in the order of carboxylate (no inhibition) < phosphate < phosphonate ≈ sulfonate < bisphosphonate < sulfate (IC_50_ ≈ 10 nM) [35]. A structure-activity relationship regarding the size and surface charge density of dPGS revealed that the L-selectin inhibition increased with both the size of the dPG core (0.24–800 kDa) at identical degree of sulfation (80%) and the degree of sulfation (10–90%) for a given core size (3 and 6 kDa) [36]. The authors concluded that the inhibition of L-selectin was caused by electrostatic interaction of dPGS to positively-charged protein motifs, as well as by steric shielding of the carbohydrate-binding site. Additionally, Paulus et al. studied the effect of dPGS branching on inflammatory processes [31]. The L-selectin inhibition efficiency of dPGS was evaluated by SPR measurements using nanoparticles with varying degree of branching (DB = 24%, 42%, 60%, and 100%). Interestingly, the dPGS with a DB of 60% revealed the highest binding affinity (IC_50_ = 2 nM), while the sulfated, perfect dendrimer (DB = 100%) gave the lowest one (IC_50_ = 300 nM).

All these findings led to the investigation of dPGS against other diseases that are associated with inflammatory events including osteoarthritis and rheumatoid arthritis. Gröger et al. studied the targeting of bone with the above-mentioned dPG-based anions [37]. Bone consists of nanometer-sized carbonated hydroxyapatite particles embedded in a collagen matrix. While the phosphate, phosphonate, and bisphosphonate functionalized polymers intensively penetrated the bone by binding to hydroxyapatite, the sulfates, sulfonates, and carboxylates bound less to hydroxyapatite but more efficiently to collagen compared to the other polyanions. The dPG-based bisphosphonate, phosphate, and sulfate as well as a mixed anion containing sulfate and bisphosphonate groups were further studied by Reimann et al. towards the interaction with native and inflamed cartilage [38]. Binding was observed for highly functionalized dPG phosphate, bisphosphonate, and sulfate, while the mixed anion showed a high affinity to cartilage. All these highly functionalized dPG anions were cytocompatible and taken up by chondrocytes. Low functionalized dPG bisphosphonate, on the contrary, was not taken up by chondrocytes but accumulated in mineralized compartments of inflamed joints and showed an enhanced affinity to cartilage with higher clinical scores. dPGS revealed a high affinity to cartilage, independent of the score, but no interaction with bone. As a result, dPGS might be a promising candidate for selective cartilage targeting.

Furthermore, Schneider et al. examined the effects of dPGS on articular chondrocytes as well as its influence on knee osteoarthritis. Since osteoarthritis progression goes along with the activation of chondrocytes and synovial fibroblasts which release pro-inflammatory cytokines such as tumor necrosis factor (TNF)α or Interleukin (IL)-6, the authors studied first the metabolic activity of chondrocytes treated with dPGS, its cellular uptake with fluorescently-labeled dPGS (dPGS-ICC), and the influence on pro- and anti-inflammatory cytokines [39]. dPGS was rapidly taken up by chondrocytes and synovial fibroblasts, but the nanoparticle did not impair the metabolic activity of chondrocytes. Incubation of full-thickness articular cartilage chips with dPGS-ICC resulted in penetration up to 50 µm in depth. dPGS treatment led to upregulation of the anti-inflammatory cytokine IL-10 but not of pro-inflammatory TNFα and IL-6. Afterwards, the authors proved the uptake of dPGS by rat-derived articular chondrocytes and showed that dPGS could modulate knee joint cartilage degradation in a rat osteoarthritis model [40]. The cytotoxicity and cellular uptake of dPGS were evaluated in rat-derived articular chondrocytes showing expectedly rapid cellular uptake but no cytotoxic effects. Subsequently, osteoarthritis was induced in the knee joints of rats. Six weeks later, rats were treated daily with dPGS and inspected for gait alterations. Finally, knee joints, liver, spleen, and kidneys were explanted and histologically analyzed. dPGS showed chondroprotective properties in the knee joints and no accumulation of dPGS in the metabolizing and excreting organs was observed. Therefore, dPGS could be a suitable drug for the treatment of osteoarthritis.

Additionally, dPGS-containing hydrogels have been applied for the treatment of osteoarthritis [41,42,43]. Hydrogels are water-swollen, cross-linked polymer networks that mimic the 3D environment of cells in native tissues. Dey et al. prepared dPGS-polyethylene glycol (PEG) hydrogels with different amounts of dPGS, characterized the hydrogel’s properties by a rheological study, and examined the chondrocyte viability in these hydrogels [41]. Among all tested hydrogels, the cell viability was higher in dPGS incorporated gels compared to PEG hydrogels (without any dPGS) which are commonly used in cartilage tissue engineering. Finally, by a little modification of the hydrogel, namely, the introduction of poly(ε-caprolactone) into the PEG-linker, Dey et al. made the hydrogels degradable [42].

Furthermore, Maysinger et al. demonstrated that dPGS could potentially be applied for the treatment of neurological disorders [44,45], which are often associated with inflammation, too. Since hyperactivity of microglia is characteristic for many neurological disorders, the authors studied the response of microglia caused by treatment with dPGS. They showed that dPGS was internalized in microglia and normalized the status of hyperactive microglia, but did not impair the cell viability. In addition, dPGS reduced the production of pro-inflammatory cytokines and did not adversely affect the structure of hippocampal dendritic postsynaptic spines. Therefore, dPGS was considered a promising candidate for the treatment of neurological disorders associated with inflammation.

## 4. dPGS as a Carrier for Anticancer Therapy

Using different cell lines, it has been shown that dPGS was rapidly taken up by cells, while cellular uptake of non-sulfated low molecular weight dPG was not observed [29,30,31,46]. Subsequently, dPGS was investigated as a potential carrier for drugs, in particular anticancer drugs. Paulus et al. studied dPGS with hydrophobic cores for the encapsulation of hydrophobic drugs [47]. Aromatic phenyl, naphthyl, and biphenyl were incorporated into the dPGS structure, and subsequently the encapsulation properties were studied with pyrene and indocarbocyanine (ICC) dye. Here, the influence of these aromatic units and their position within the polymer scaffold was examined by comparison of statistical and block copolymers. Two of these architectures revealed high transport capacities (in the low micromolar range), namely, the block copolymers with naphthyl and biphenyl groups in the core indicating that a core-shell type architecture was more suitable for the transport of guest molecules than a statistical distribution of the hydrophobic units. However, none of the investigated compounds were further applied as carriers for drug encapsulation.

At the same time, Sousa-Herves et al. conjugated paclitaxel (PTX) chemically to dPGS via an acid-labile ester linkage, and the resulting conjugate was characterized and tested in vitro [32]. Paclitaxel release at different pH values was determined by high-performance liquid chromatography (HPLC), the cellular uptake was conducted by flow cytometry and confocal microscopy, and the cytotoxicity was evaluated. The conjugate was successfully taken up by cancer cells and had a cytotoxic effect on these cells, but it demonstrated poor stability in plasma and at physiological pH, which resulted in premature drug release. To overcome this limitation and to improve the therapeutic properties of dPGS-PTX, Ferber et al. used the pH-cleavable but more stable hydrazone bond for conjugating PTX to dPGS for the treatment of glioblastoma, which is the most common kind of brain tumor and is one of the most aggressive forms of cancer [48]. The authors have shown that dPGS could target the tumor microenvironment as well as the cancer cells themselves, via binding to P-selectin expressed on glioblastoma cells. The hydrazone linker enabled the successful release of PTX into glioblastoma, which led to significantly inhibited tumor growth, while circumventing the side effects related to PTX.

Alternatively, Zhong et al. investigated doxorubicin (DOX)-loaded, biodegradable micelles with sheddable dPGS shells that showed extraordinary tumor targetability and chemotherapy in MCF-7 human mammary carcinoma-bearing mice [49]. The micelles based on self-assembled dPGS-SS-poly(ε-caprolactone) amphiphilic block copolymers, revealed low cytotoxicity, high DOX loading, low drug leakage, and accelerated drug release under reductive conditions. In vivo studies with DOX-loaded micelles showed a high tolerated dose, and long plasma circulation time. Furthermore, they caused complete inhibition of tumor growth, markedly improved survival rates, and fewer adverse effects than the free drug (DOX·HCl).

## 5. dPGS as an Antiviral Agent

Over the last few decades, polyanionic compounds, particularly polysulfates, have gained increasing attention due to their inhibitory effect on a variety of enveloped viruses [50,51,52]. Recently, dPGS-functionalized gold nanoparticles and graphene sheets were applied for virus inhibition. Vonnemann et al. studied the inhibition of vesicular stomatitis virus by dPGS-functionalized gold nanoparticles and found that the virus inhibition was dependent on the nanoparticle size as well as the contact area between the virus and the nanoparticle [53]. Particles with similar or larger sizes than the virus inhibited the virus-cell binding and thus the infection more efficiently than smaller ones.

Using dPGS-functionalized graphene sheets, Ziem et al. demonstrated the excellent binding and efficient inhibition of orthopoxvirus infection as well as African swine fever virus and the alphaherpesvirus pseudorabies virus causing Aujeszky’s disease in pigs indicating that these new multivalent 2D polymer nanosystems could interact with various types of viruses, prevent viral adhesion to the host cell and especially target viruses that rely on a heparan sulfate-dependent cell entry mechanism [54,55].

## 6. Labeled dPGS for Diagnostics

**Figure 4:**
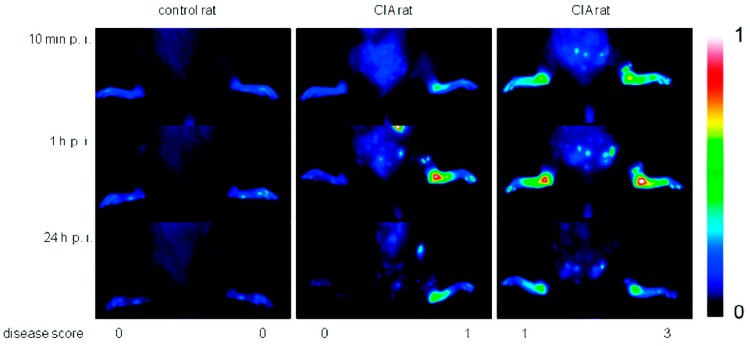
Comparison of fluorescence images in false colors (normalized to a fluorescence reference cube) of a control rat and rats with collagen-induced rheumatoid arthritis (different clinical scores are indicated) after 10 min, 1 h, and 24 h post injection of 6 (4 mg/kg b.w.). One representative example of at least *n* = 5. Reprinted with permission from [29]. Copyright 2011 American Chemical Society.

Due to their high affinity to inflamed tissue, dPGS has been successfully applied in diagnostics. Licha et al. examined the targeting of inflammation with dye-labeled dPGS in vivo [29]. Using near-infrared (NIR) fluorescence imaging in an animal model of collagen-induced rheumatoid arthritis, the authors showed that dPGS accumulated in diseased joints (Figure 4), whereas non-sulfated dPG was not detected in association with the disease. Furthermore, Biffi et al. used the dPGS-NIR dye conjugate to detect inflammatory reactions within the lungs, as shown in mice with acute allergic asthma [46]. These in vivo studies indicated that the dPGS-NIR dye conjugate could probably be used to monitor inflammation processes and responses to therapy.

The same dPGS-dye conjugate has been used for visualization of myocardial infarction and rheumatoid arthritis by optoacoustic imaging, particularly multispectral optoacoustic tomography (MSOT) [56,57]. While common optical imaging methods lack high spatial resolution and thus accuracy at increased penetration depths, with optoacoustic imaging methods images with high resolution can still be obtained from tissues up to several centimeters deep. After Taruttis et al. had detected dPGS-NIR in infarcted myocardium in mice [56], Beziere et al. used the dPGS-NIR conjugate and MSOT in a murine arthritis model to visualize the extent of inflammation in vivo [57]. This method allowed an accurate diagnosis of inflammation in the mouse joints and showed promise for diagnosing rheumatoid arthritis and the staging of arthritis-related inflammation.

In order to study the localization and distribution of dPGS in vitro as well as in vivo, different dye- and radio-labeling strategies were investigated. Cyanine dyes were conjugated to dPGS and the resulting dye-labeled conjugates were studied by fluorescence and fluorescence life-time imaging [29,30,58,59]. Furthermore, ^35^S, ^3^H, and ^64^Cu radioisotopes were incorporated in the dPGS structure [60,61,62]. Accumulation of dPGS in liver and spleen was observed three weeks after intravenous administration in mice and rats [30,62], which might limit the applicability of bioactive dPGS as a therapeutic agent. To avoid undesired organ accumulation of dPGS, Reimann et al. investigated different shell-cleavable dPG sulfates, namely, dPG-amidoglyceryl succinyl sulfate (dPG-ASuS), dPG-thioglyceryl pentanoatyl sulfate (dPG-TPS), and dPG-thioglyceryl methylpropanoatyl sulfate (dPG-TMPS) with similar anti-inflammatory activity compared to non-degradable dPGS [63]. Therefore, these shell degradable polysulfates could potentially be applied for the long-term treatment of chronic inflammation as well as in tissue engineering due to their high anti-inflammatory but low anticoagulant properties. Since dPG-TMPS and dPG-TPS notably reduced the complement activation, the authors claimed that these scaffolds could also be considered as a new class of anticomplement therapeutics.

## 7. Conclusions

Our review summarizes the potential applications of dPGS as a synthetic heparin analog with high inflammation targeting ability suitable for imaging, diagnosis and treatment of different diseases. Originally investigated as a synthetic heparin analog, dPGS showed much less anticoagulant activity, but high anti-inflammatory activity. Due to the ability to target inflamed tissue, dPGS could be used to diagnose and treat rheumatoid and osteoarthritis. Furthermore, dPGS is taken up by tumor cells and able to target tumor tissue, which makes it a suitable carrier for anticancer drugs. Last but not least, dPGS-functionalized gold nanoparticles and graphene sheets could be used for the treatment of virus infections as they interact with different virus types. In summary, it can be stated that dPGS possesses the potential to open up promising applications in the field of nanomedicine and diagnostics.
